# Knowledge and experiences of adolescent girls and young women in the use of sexual reproductive health and HIV services at health facilities in Maputo City, Mozambique

**DOI:** 10.3389/frph.2025.1667930

**Published:** 2025-11-20

**Authors:** Vasco Muchanga, Luisa Huo, Kathryn T. Kampa, Baltazar Chilundo, Khátia Munguambe, Troy D. Moon

**Affiliations:** 1 Department of Community Health, Faculty of Medicine, Eduardo Mondlane University, Maputo, Mozambique; 2 Department of Tropical Medicine and Infectious Diseases, School of Public Health and Tropical Medicine, Tulane University, New Orleans, LA, United States

**Keywords:** adolescent, adolescent girls and young women, sexual reproductive health, HIV, Mozambique

## Abstract

**Introduction:**

Knowledge and use of sexual reproductive health and human immunodeficiency virus (SRH and HIV) services are crucial for the prevention of pregnancy and sexually transmitted infections (STIs) among adolescent girls and young women (AGYW). This study aims to evaluate the knowledge and perceptions of AGYW about the SRH and HIV services offered in health facilities in Maputo, Mozambique.

**Methods:**

An analytical cross-sectional study was conducted based on exit surveys with AGYW held at the health facilities named Zimpeto and June 1st, in Maputo City, between May 1, and June 9, 2023. Data were analyzed through descriptive statistics, t-test and ANOVA, using SPSS version 20. 590 AGYW, aged 15–24 years of age, were included in the study.

**Results:**

In general, knowledge of SRH and HIV services was fairly high, with knowledge of each specific service offered ranging between 38% and 97%. Knowledge about SRH and HIV services differed depending on the health facility where the AGYW sought SRH and HIV services; the participant's age; their occupation; their religion, and who they lived with. Counseling services were the most commonly reported services attended, with >90% of participants reporting having received counseling for each of the following: STI and HIV and pregnancy prevention and safer sex practices. The quality of SRH and HIV services, as well as the attitudes of the providers were considered to be good by >90% of AGYW. Roughly 95% of AGYW at Zimpeto health facility, were either “satisfied” or “very satisfied”. Whereas at the June 1st health facility, only roughly 75% of AGYW were either “satisfied” or “very satisfied”, and roughly 20% of AGYW were “little satisfied” that their needs had been met that day.

**Discussion:**

Among AGYW interviewed there is a high level of knowledge about SRH and HIV counseling services as compared to STI testing, treatment, and clinical care. Specific attention should be given to ensuring appropriate physical infrastructure, such as dedicated adolescent friendly spaces and comfortable seating. Targeted interventions that are focused on improving the quality of the services delivered, should be designed and implemented for those health facilities perceived by AGYW to have lower quality.

## Introduction

Adolescent girls and young women (AGYW) remain at the highest risk of acquiring human immunodeficiency virus (HIV) in sub-Saharan Africa (1). Globally in 2023, it was estimated that an average of 4,000 AGYW aged 15–24 years became newly infected with HIV each week, of which roughly 75% occurred in sub-Saharan Africa. AGYW are three times as likely to acquire HIV than their male counterparts (2).

Evidence shows that in sub-Saharan Africa, adolescents engage in sexual activity at a very young age (average age of 13 years), yet most do not use any form of protection against unintended pregnancies or sexually transmitted infections (STIs) (3). In a multi-country analysis, the prevalence of first pregnancy among AGYW ranged from 7.2% (in Rwanda) to 44.3% (in the Democratic Republic of Congo) (4). General knowledge about STIs, including HIV, as well as about sexual reproductive health (SRH) services has been shown to be limited among AGYW (5). Further, AGYW face a number of challenges in terms of access to comprehensive health care, meeting their contraceptive needs, and the ability to negotiate safe sex (6).

Despite various efforts by the Mozambican Ministry of Health (MoH) to promote adolescent and youth-friendly health services (AYFHS) aimed at preventing STIs, including HIV, and early pregnancy, Mozambique has the highest percentage of AGYW who initiated sexual intercourse before the age of 15 compared to other countries in sub-Saharan Africa (7, 8). Furthermore, Mozambique ranks third in terms of countries with the highest birth rate among adolescents (123 per 1,000 women) (8). In 2023, 61% of females in Mozambique reported having had a live birth by the age of 19 years. Mozambique is also a country with a high HIV disease burden. In 2023, the HIV prevalence ranged from between 8 and 21% across Mozambique's 11 provinces. In the same year, Maputo, the country's capital city, reported an HIV prevalence of 16%. Nationwide, HIV prevalence among persons aged 15–24 years, stands at 5.4%. Within this age group, women have a higher HIV prevalence (8%) as compared to men (2.6%). While 54% of AGYW have been reported as having tested for HIV, only 1.4% received their results, which could give rise to gaps in the provision of HIV services (9).

A 2017 analysis of the gaps and obstacles in priority interventions for the prevention and treatment of HIV/AIDS in adolescents in Mozambique, found that <40% of adolescents reported having accessed AYFHS, and only 13.6% reported actively using a modern contraceptive method (10). The most recent Demographic Health Survey (2023) carried out in Mozambique, indicates that the prevalence of modern contraceptive use was approximately 16% among AGYW (11).

Studies on the barriers to accessing SRH and STIs, including HIV services across sub-Saharan Africa, commonly report inadequate information on the part of the AGYW about the availability of services, as well as their misperceptions about SRH and HIV services. In addition, services offered in an unsupportive environment and poor provider attitudes have been listed as potential barriers to AGYW accessing care (12, 13).

Although the literature on AGYW's knowledge of SRH in Mozambique is limited, existing studies highlight significant gaps. A study in Nampula Province found that AGYW generally had accurate knowledge about HIV, including condom use as a preventive method (14). Another study conducted in the same province revealed that less than half of AGYW (42%) were informed about the benefits of family planning, 46% knew about three different types of contraceptives, and only 29% were aware of potential side effects (15). However, the 2022/23 National Demographic and Health Survey reported even lower HIV prevention knowledge among AGYW (28.01%) compared to their male peers (32.03%). Collectively, these findings underscore the need for targeted interventions and further research to improve AGYW's awareness of SRH, and STIs, including HIV services (11).

To contribute to addressing gaps in the understanding of AGYW utilization of services for SRH, STIs, and HIV in Mozambique, this study aimed to first, assess AGYW knowledge about SRH, and STIs, including HIV service availability, and second, to explore AGYW experiences with utilizing SRH and HIV services at two health facilities in Maputo City, Mozambique. In this study, SRH, STI, and HIV services are part of a package of services offered through the AYFHS in public health centers across Mozambique. This package is outlined in the Guideline for the Implementation of AYFHS in Health Facilities, Schools, and Communities (16), and aligns with the National School Health and Adolescent and Youth Health Strategy 2019—2029 (17), and the WHO 2017 recommendations on adolescent health (18) and adolescent sexual and reproductive health and rights (19). This package of services includes: “information, education, and communication” services on SRH topics of relevance to adolescents; counseling and testing for STI and HIV; breast and cervical cancer screening; family planning, including the provision of intrauterine devices (IUD); antenatal, delivery, and postnatal care; management of uncomplicated post-abortion cases, including counseling and referral for complicated cases; comprehensive HIV care and treatment for adolescents and young people living with HIV, including differentiated service delivery models adapted to AYFHS; as well as psychosocial support for people living with HIV and referral to support groups (16). For the purpose of this study, these services have been categorized into the following areas: sexuality counseling; safe-sex counseling; pregnancy-prevention counseling; counseling on STIs, including HIV prevention; pregnancy testing; STI and HIV diagnostic testing; gender-based violence services; antenatal care; and postnatal care.

## Materials and methods

### Study design

We conducted an analytical cross-sectional study to evaluate AGYW knowledge about, and experiences with, accessing SRH, and STIs, including HIV services in Mozambique. This analysis represents baseline (phase one) data collection of a larger multi-phase implementation science study that utilized a mixed-method approach, aiming to assess the feasibility and effectiveness of an “adolescent-friendly approach” for improving access to and use of SRH, and STIs, including HIV services by AGYW at selected health facilities. At baseline, we conducted an exit survey with AGYW who had sought SRH services, STI, and including HIV services, within AYFHS at two primary health care level facilities: Zimpeto and June 1st health facilities (*Centro de Saúde 1 de Junho*) in Maputo City, Mozambique. AGYW primarily sought SRH services related to family planning, maternal health, STI care, HIV testing and counseling, antiretroviral therapy, and health education. These centers were selected due to their historically poor performance in providing AGYW with access to and utilization of SRH and HIV services.

### Study population

All AGYW, aged 15–24 years, who had accessed SRH and HIV services at either of the two study centers between May 1 and June 9, 2023, were considered eligible to participate in the exit survey. AGYW were selected by convenience and approached for enrollment as they were exiting the health center (provided they had contact with the SRH or HIV services on the day of the interview). Initially, a sample size of 520 AGYW was defined, but during the data collection process, 598 were consented and enrolled. The sample size was calculated using the following formula: $n=\frac{\;p(1\;-\;p)Z^{2}}{\varepsilon^{2}}$; n: sample size; p: expected proportion; z: value of the normal distribution for a given confidence level, and *ε* = size of the confidence interval. Where *p* = 0.38 (10); z = 1.645; ε=0.05. A 95% confidence interval was used for a 5% significance level. The value of *n* was doubled, taking into account that data collection would take place in two health centers.

### Data collection and management

Interviews took place on weekdays, during normal service hours (7:30 a.m. to 3:30 p.m.). The first AGYW to leave the health facility during this time interval was approached and, if accepted, she was interviewed. Only after the interview was finished would subsequent participants be approached for recruitment. This continued until the defined sample was reached. Trained interviewers conducted the interviews using a semi-structured interview guide in a private location. The interviews were conducted in Portuguese.

AGYW were questioned about their sociodemographic data; knowledge of the types of SRH and STIs, including HIV services offered at the facility; types of SRH and, STIs, including HIV services received on the day of the visit; the perceived quality of the SRH and HIV services offered on the day of the visit; and their level of satisfaction with the SRH and HIV services received on the day of the visit (Supplementary File S1). Questions designed to assess AGYW knowledge about the services offered and received, were based off a list of SRH and HIV services outlined in the Guidelines for the Implementation of adolescent and youth-friendly health services of the Mozambican Ministry of Health (16)*.*Questions related to the perceived quality of the services offered were based on World Health Organization (WHO) standard quality assessment questions for AYFHS (20).

A survey tool was developed using REDCap (Research Electronic Data Capture) and uploaded onto a tablet computer. During the interview, interviewers input data directly into the tablet (REDCap v10.6.12, 2023). At the end of each day, data was synchronized and sent to a REDCap server housed at the Faculty of Medicine of University Eduardo Mondlane (UEM) in Maputo. The data were extracted from REDCap into excel format, and then imported into the Statistical Package for the Social Sciences (SPSS) Version 20. Data were cleaned and all lines with missing data for reference variables for analysis were excluded from the analysis. Sociodemographic variables with a variety of different categories were aggregated in order to simplify analysis. The aggregated variables include: Age—Transformed into two age ranges: “15–19 years”, and “20–24 years”; Who do you live with?; the responses “grandmother”, “grandfather” and “grandparents” were combined into a single category called “Grandparents”; the responses of father, mother, parents were aggregated into a single category called Parents; and Religion: All Christian responses of non-Catholic religious affiliation have been aggregated into the Protestant category. To assess the average knowledge of AGYW about the SRH services offered at the two health facilities, participants were asked to answer 10 questions. Questions were given a score of either 0 or 10; 0 if the response was wrong and 10 if it was correct. Therefore, the 10 questions had a minimum total score of 0 (if no question was answered correctly) and a maximum of 100 points (if all questions answered correctly). The same scale was converted into a percentage, and a scale of 0 to 100% was adopted. All the questions were given the same weight.

### Data analysis

Descriptive statistics, including absolute and relative frequencies, were used to determine which SRH and HIV services were known to AGYW, which services were utilized (including modern contraceptive methods) and to evaluate AGYW's perceptions of the health facility environment for adolescent care. The One-Way Anova (Analysis of Variance) test was applied to test the equality of the means of knowledge of SRH, and STI, including HIV services in AGYW. The student's t-test was applied to compare the means of AGYW 's knowledge**.** Level of knowledge of SRH, and STIs, including HIV services, was checked for normality using the Shapiro Walk test. The result showed that it does not follow a normal distribution (*p* < 0.001). Although it does not follow a normal distribution, the variable shows low variability (mean = 81.29 ± 21.379) and a coefficient of variation (CV) equal to 26%. A 95% confidence interval was used for a 5% significance level, α = 0.05.

### Ethical considerations

The study protocol was reviewed and approved by the Mozambican National Bioethics Committee for Health (Ref: 88/CNBS/23). Administrative approval was granted by the MoH of Mozambique (Note nr: 396/GMS/290/023). All participants provided written informed consent prior to participation in the exit interview. For participants below the age of 18 years, informed consent was first obtained from a parent/guardian and then informed assent from the participant. No identifying information was recorded by the interviewer to ensure anonymity.

## Results

### Participant characteristics

A total of 598 AGYW were approached and surveyed. Eight participants were subsequently excluded from the analysis because of missing data. A total of 590 AGYW were included, of which 304 (51.5%) were recruited from the June 1st health facility and 286 (48.5%) from the Zimpeto health facility. Fifty-one percent of AGYW were aged 15–19 years and 49% were aged 20–24 years, with a mean age of 20 years ±2.42. Approximately 70% (*n* = 419) of respondents listed themselves as a current student, of which the majority (86.8%) had achieved at least some level of secondary education. Of the AGYW interviewed, 79.6% (*n* = 468) were single, while 20.4% (*n* = 122) reported being in a marital union. The majority of participants (65.5%) lived with their parents, while 19.3% (*n* = 114) lived with their husbands. Protestant was the leading religious affiliation (53.5%), followed by Catholic (34.5%), and Muslim (4.9%) (Table 1).

**Table 1:** Participant sociodemographic characteristics.

Characteristic (*n* = 590)	*n* (%)
Health centers	
1° de Junho	304 (51.5)
Zimpeto	286 (48.5)
Age	
15–19 years	301 (51.0)
20–24 years	289 (49.0)
Mean age	20 ± 2.42
Level of education	
No education	27 (4.6)
Primary	31 (5.3)
Secondary	513 (86.8)
Higher	19 (3.2)
Occupation	
Student	414 (70.2)
Unemployed	176 (29.8)
Marital status	
Marital Union^a^	122 (20.4)
Single	468 (79.6)
Who do you live with?	
Alone	6 (1.0)
Parents	386 (65.4)
Siblings	19 (3.2)
Grandparents	30 (5.1)
Uncles	35 (5.9)
Husband	114 (19.3)
Religion	
No religion	42 (7.1)
Protestant	316 (53.5)
Catholic	203 (34.4)
Muslim	29 (4.9)

a Marital Union = informal marriages.

### AGYW knowledge of sexual reproductive health and HIV services available at health facility

**Figure 1:**
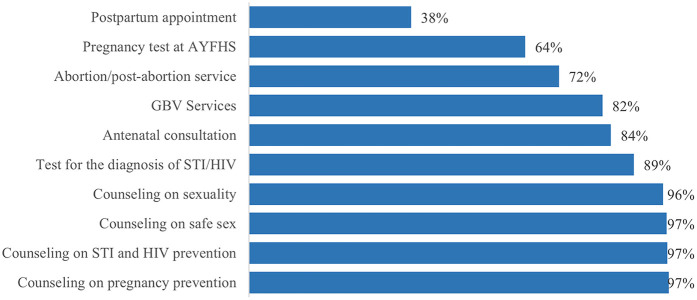
Knowledge of AGYW about SRH and HIV services offered.

In general, AGYW knowledge of SRH and HIV services was fairly high, with knowledge of each specific service offered ranging between 38% and 97%. The best-known SRH and HIV services were pregnancy prevention counseling (97%); HIV and STI prevention counseling (97%); safe sex counseling (97%); sexuality counseling (96%); HIV and STI diagnostic testing (89%); antenatal care consultations (84%); and gender-based violence (GBV) services (82%). Pregnancy testing services (64%), and abortion services (72%) were moderately known, and the least known service was the postpartum care clinics (38%) (Figure 1).

Average knowledge about SRH and HIV services differed depending on where the AGYW sought healthcare, the participants age, their status of employment, their religion, and who they lived with. AGYW who attend the Zimpeto health facility had a higher average level of knowledge about the SRH and HIV services offered (86.6%; CI: 83.8–89.3), compared with those who attended the June 1st health facility (76.3%; CI: 74.3–78.3) (*p* < 0.001). AGYW aged 20–24 years had a higher average level of knowledge (83%; CI: 80.7–85.4) when compared to AGYW aged 15–19 years (79.6%; CI: 77.1–82.1) (*p* = 0.046). AGYW who are actively in school had a higher average level of knowledge about SRH and HIV services (82.4%; CI: 80.2–84.5), compared to adolescents who are out of school (78.7%; CI: 75.8–81.7) (*p* = 0.048). AGYW who reported a religious affiliation: Protestant (77.9%; CI: 75.5–80.3), Catholic (86.5%; CI: 83.8–89.3), and Muslim (88.9%; CI: 83.2–94.7), had a higher average level of knowledge as compared to those reporting no religious affiliation (75.8%; CI: 67.9–83.8) (*p* < 0.001). Of note, a significantly higher average level of knowledge was observed among Muslim and Catholic participants, compared to Protestants or non-religious counterparts. Lastly, AGYW who live with their parents had a higher average level of knowledge (84.20% CI: 82.2–86.2) about SRH and HIV services as compared to participants in other living arrangements (*p* < 0.001) (Table 2).

**Table 2:** Factors associated with knowledge about SRH and HIV services offered .

Characteristics	*n*	Mean	95% CI	*p-value*
Health facility				<0.001
June 1st	304	73.3	74.3–78.3	
Zimpeto	286	86.6	83.8–89.3	
Age				0.046
15–19 years	301	79.6	77.1–82.1	
20–24 years	289	83.1	80.7–85.6	
Level of education				0.089
No schooling	27	83.7	72.7–94.7	
Primary	31	72.9	63.9–81.9	
Secondary	513	81.9	80.0–83.7	
Higher	19	76.3	70.5–82.2	
Occupation				0.048
Student	414	82.4	80.3–84.5	
Unemployed	176	78.7	75.7–81.7	
Marital status				0.118
Single	468	81.8	79.9–83.8	
Marital Union	117	78.4	74.8–82.0	
Religion				<0.001
No religion	41	75.9	67.9–83.8	
Protestant	316	77.9	75.6–80.3	
Catholic	203	86.6	83.8–89.3	
Muslim	29	89.0	83.2–94.8	
Who do you live with?				<0.001
Alone	6	78.3	66.1–90.6	
Parents	386	84.2	82.2–86.2	
Siblings	19	81.6	74.2–89.0	
Grandparents	30	69.3	57.8–80.9	
Uncles	35	69.7	60.9–78.5	
Spouse	114	78.3	74.5–81.9	

### Types of SRH and HIV services received by AGYW on the day of the health facility visit

**Figure 2:**
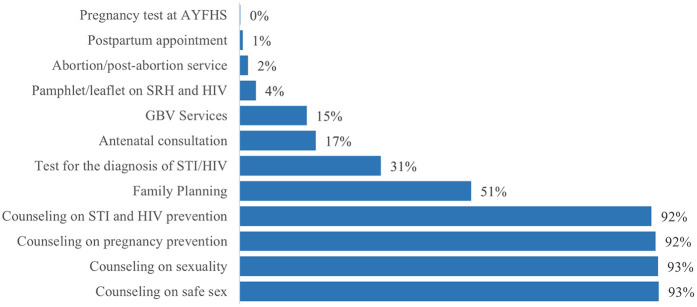
Types of SRH and HIV services received by the AGYW on the day of visit to the health facility.

AGYW reported a variety of SRH and HIV services they sought on the day of their interview. Counseling services were the most commonly reported services, with >90% of participants reporting having received counseling for each of the following: STI and HIV prevention, pregnancy prevention, sexuality, and safer sex practices. Further, 51% of participants attended family planning services and 31% received testing for HIV and/or another STI. Smaller numbers of participants attended other services such as antenatal care or gender-based violence (GBV) counseling (<20% for each service) (Figure 2). Among those participants who attended family planning services on the day of their interview (*n* = 303), birth control options were received in the following proportions: 33% injectables (Depo-Provera); 30% oral contraceptive pills; 16% male condoms; 12% female condoms; and 10% implant. Participants could receive more than one type of contraceptive.

### AGYW perceptions of the quality of SRH and HIV services offered at their health facility

Overall, more than 90% of participants reported to have received counseling in private spaces and that upon arrival to the service, they were greeted and served according to their needs. At Zimpeto health facility, over 95% of participants reported that services were offered in a dedicated space catering to adolescents. In contrast, only roughly 13% of participants at the June 1st health facility reported a lack of an adolescent dedicated space. In addition, the vast majority of participants (87.8%) at the June 1st health facility reported a lack of separate waiting room for adolescents, compared to Zimpeto health facility, where <10% of participants reported a lack a separate waiting room. Overall, only 4% of AGYW correctly reported that the health facility had a dedicated schedule for when adolescent services were available. Of the other respondents, 54.2% responded that the facility did not have a dedicated schedule and 41.7% responded that they did not know. At both facilities, >25% of AGYW reported no comfortable sitting arrangements at the waiting area.

In terms of service quality, >90% of participants at both facilities ranked the attitude of the provider as “Good”. With regards to service quality, >90% of participants at Zimpeto ranked the services as “Good”, whereas at June 1st, only 79% ranked the services as “Good” and 18% ranked them only as “Acceptable”.

When asked about how satisfied the participant was with regards to whether their needs had been met that day, roughly 95% of AGYW at Zimpeto health facility responded that they were either “satisfied” or “very satisfied”. In contrast, at the June 1st health facility, only about 75% of AGYW reported that they were either “satisfied” or “very satisfied”, with another 20% responding that they were “little satisfied” (see Table 3).

**Table 3:** AGYWs’ experiences and perceptions about SRH and HIV services offered.

How do you evaluate the environment of the health facility's services to adolescents?		June 1st *n* (%)	Zimpeto *n* (%)	Total *n* (%)
Is there a specific schedule for adolescents in this health facility?	Dont Know	154 (50,7)	92 (32,2)	246 (41,7)
	No	127 (41,8)	193 (67,5)	320 (54,2)
	Yes	23 (7,6)	1 (0,3)	24 (4,1)
Does the health facility have a comfortable place for adolescents to sit?	Dont Know	1 (0,3)	1 (0,3)	2 (0,3)
	No	108 (35,5)	68 (23,8)	176 (29,8)
	Yes	195 (64,1)	217 (75,9)	412 (69,8)
Does the health facility have a separate space to offer services to adolescents?	Dont Know	8 (2,6)	6 (2,1)	14 (2,4)
	No	40 (13,2)	8 (2,8)	48 (8,1)
	Yes	256 (84,2)	272 (95,1)	528 (89,5)
Does the health facility have a separate waiting room for adolescents?	Dont Know	9 (3,0)	3 (1,0)	12 (2,0)
	No	267 (87,8)	19 (6,6)	286 (48,5)
	Yes	28 (9,2)	264 (92,3)	292 (49,5)
Is there a counseling area for adolescents that provides privacy?	Dont Know	2 (0,7)	0 (0,0)	2 (0,3)
	No	21 (6,9)	5 (1,7)	26 (4,4)
	Yes	281 (92,4)	281 (98,3)	562 (95,3)
Are adolescents greeted and served according to their needs or those of their partner(s)?	Dont Know	1 (0,3)	0 (0,0)	1 (0,2)
	No	24 (7,9)	4 (1,4)	28 (4,7)
	Yes	279 (91,8)	282 (98,6)	561 (95,1)
How do you evaluate the attitude of the provider who assisted you?	Bad	10 (3.3)	0 (0.0)	10 (1.7)
	Acceptable	40 (13.2)	9 (3.1)	49 (8.3)
	Good	254 (83.6)	277 (96.9)	531 (90.0)
Overall, how do you evaluate the provision of SRH and HIV services in this health facility?	Bad	9 (3.0)	3 (1.0)	12 (2.0)
	Acceptable	55 (18.1)	25 (8.7)	80 (13.6)
	Good	240 (79.0)	258 (90.2)	498 (84.4)
How satisfied were you that your needs were met today?	Little satisfied	62 (20.4)	12 (4.2)	74 (12.5)
	Not satisfied	17 (5.6)	3 (1.2)	20 (3.4)
	Satisfied	193 (63.5)	258 (90.0)	451 (76.4)
	Very satisfied	32 (10.5)	13 (4.6)	45 (7.7)

## Discussion

This study aimed to evaluate the knowledge and perceptions of AGYW seeking health services at selected health facilities in Maputo, Mozambique, regarding the available SRH and HIV services, and to explore their experiences in accessing and utilizing these services. This type of assessment is relevant and addresses the WHO recommendation to ensure that adolescents are aware of what health services are being provided, and where, when, and how to obtain them (20).

Broadly, knowledge about SRH and HIV services in our population of Mozambican AGYW was high. Specifically, the best-known services were counseling services at the health centers related to safe sexual practices including pregnancy prevention, STI, including HIV prevention, and understanding one's sexuality. Awareness of testing services for pregnancy and STI, including HIV was slightly less, and the least known services were those related to antenatal and post-partum care. When we compare our AGYW population to other similar populations across sub-Saharan Africa, our findings show that generalized knowledge about SRH and HIV services in Mozambican AGYW is higher (21–24). However, when we begin looking at the different types of services individually, we found that knowledge of HIV and STI testing was generally higher in Mozambique compared to other countries such as Ghana, Ethiopia, and Nigeria (21, 25, 26). The high level of knowledge observed in our study, compared to those carried out in Ghana, Ethiopia, and Nigeria, may be linked to the fact that our study recruited from a pool of AGYW that had just had contact with SRH and HIV services. Whereas the studies carried out in other contexts were based at community level, possibly with a memory bias, which can limit the level of knowledge of SRH and HIV services. When looking at knowledge of GBV services, Mozambican AGYW had relatively poor knowledge, which was similar to what was reported in these other countries (15, 19, 20).

We found a significant association between knowledge score and increased age, being a student, religion, the health facility where services were sought, and whom one lives with. These findings are not surprising. First, with increased age, AGYW gain the autonomy to make personal decisions about their health. Its likely that older AGYW have had more opportunities to visit a health facility and have had more life experiences that could enhance their overall knowledge about these services. Second, AGYW who attend school are more likely to have greater knowledge than those who do not, as schools are a primary source of health information (25–28). There is controversial evidence about the association between religious affiliation and the level of knowledge about SRH and HIV services. While other studies suggest that religious affiliation is a protective factor against the risk of HIV (29), there is recognition that religion may offer inadequate information related to SRH and HIV (30), and at times has been shown to have a negative influence on the level of knowledge about SRH among adolescents (31). Our results show a significant difference in the level of knowledge of SRH and HIV services for those AGYW who attend the Zimpeto health facility compared to those who the June 1st health facility. This suggests the need to delve deeper into the internal factors of each facility to better explain these differences.

Exposure to information about SRH services has been reported as one of the predictors of SRH utilization (21, 25, 32). However, in this study, this seemed to be the opposite. Among the AGYW who visited health facilities and had access to SRH counseling services, few of them had access to STI, HIV and early pregnancy prevention supplies. Similar results related to low use of HIV testing services, STI treatment, and family planning among AGYW was found in another studies conducted in similar context (8, 21, 26, 27, 33, 34).

Study participants were generally favorable about the quality of the SRH and HIV services they had accessed in terms of existence of separate and visible areas for AGYW, and the attitude of the providers they had seen. Our results, with regards to respondent perception of the quality of SRH and HIV services, were similar to another multi-country study which included Ethiopia, Nigeria, and Mozambique (15, 35, 36). However, our results contrasted with other studies carried out in Mexico (37) and Nigeria (38), which reported negative provider attitudes characterized by judgment of AGYW when they seek SRH and HIV services.

Our results suggest that the conditions of the waiting rooms at the health facilities were not comfortable. Further, there was evidence of the lack of a specific schedule for when adolescent services are offered. Nevertheless, the guide to implementing a standards approach to improving the quality of health services for adolescents, recommends that adequate seating should be made available in the waiting room for the normal flow of patients. It also recommends that health facilities should have convenient hours of operation that facilitates adolescent's access to these health services (20).

Despite these complaints, the high level of reported satisfaction among the participants seems to indicate that the quality of the service provided for AGYW is perceived as favorable. A similar high level of satisfaction was found in assessments of AYFHS from both Eastern and the southern African region (8, 15, 39, 40).

### Implications

This study has important implications for policy, research, and practice. From a policy perspective, the gap between knowledge of counselling services and awareness of diagnostics, treatment, and clinical care highlights the need for balanced dissemination of comprehensive SRH and STI (including HIV) services for AGYW to improve knowledge and service uptake. For research, findings indicate the need to examine SRH and STI (including HIV) knowledge among AGYW and adolescent boys and young men through community-based surveys with larger, geographically diverse samples. In practice, AYFHS health providers can use these findings to strengthen service dissemination at facility and community levels, enhancing knowledge and uptake of care among adolescents and young people.

### Limitations

Despite the contributions of this study, several limitations should be acknowledged. The sample may not represent all adolescents and young people in the region. Self-reported knowledge may introduce response bias, and the cross-sectional design limits causal inference. The fact that it involved only AGYW with previous contact with SRH and HIV services could be considered a possible bias, as contact with these services would presumably increase one's knowledge about them. However, targeting recruitment to AGYW immediately following their use of these services, was intended to reduce memory bias and to better capture the respondent's opinions as to their experiences in near real-time.

### Recommendations

Future research should include larger, more diverse samples, including adolescent boy and young men, and consider longitudinal or mixed-method designs to explore causal links between knowledge and service uptake. Recognizing the financial burden that comes with such methodology, with the increase on the availability of cell phones, especially in urban areas, cell phone surveys can be a better option then health facility-based surveys. Objective measures of knowledge and service use are recommended to reduce bias. Policymakers and practitioners should ensure equitable and comprehensive dissemination of SRH and STI (including HIV) services for adolescents and young people.

## Conclusion

Knowledge about SRH, and STIs, including HIV services offered in health facilities is inconsistent across the different types of services, with high levels of knowledge about counseling in contrast to diagnostics, treatment and clinical care. The results of this study suggest the need to balance the dissemination of the different SRH, and STI, including HIV services targeting AGYW in the catchment areas involved in the study. Additionally, site specific attention should be given to ensuring appropriate physical infrastructure exists that takes into account the unique needs of AGYW, such as dedicated adolescent friendly spaces and comfortable seating. Finally, targeted interventions should be designed and implemented for those health facilities, such as the June 1st health facility, which consistently perform poorer with respect to AGYW perceived quality of services offered.

## Data Availability

The datasets presented in this study can be found in online repositories. The names of the repository/repositories and accession number(s) can be found below: https://deposit.icpsr.umich.edu/deposit/workspace?goToPath=/ddf/220224&goToLevel=project.
