# Supplementary material for: Knowledge and experiences of adolescent girls and young women in the use of sexual reproductive health and HIV services at health facilities in Maputo City, Mozambique
Source: Front Reprod Health. 2025 Nov 20;7:1667930. doi: 10.3389/frph.2025.1667930 (PMC12675394; doi:10.3389/frph.2025.1667930)
Supplement: Supplementary file 1 [file Datasheet1.pdf]

UNIVERSIDADE EDUARDO MONDLANE

Faculdade de Medicina

Feasibility and effectiveness of the "adolescent-friendly" approach in accessing and using SRH and HIV services by adolescent girls and young woman in Maputo City
Questionnaire for exit survey of adolescent girls and young woman at health facility level
Version 001 of May 10, 2022
(Translated from portuguese to English)

SOCIODEMOGRAPHIC DATA:

**This section must be completed before starting the interview.**	
1. Interview date	|___|___| -|___|___|___|___| - |___|___| dd mmm yy
2. Set the start time for the interview	Hours____: Minutes_____
3. City	□ City of Maputo
4. District	4.1. Kamubukwana 4.2. Kamavota
	4.1.1 Zimpeto HF 4.2.1. June 1st HF
5. Residential Neighborhood	
**This section must be completed during the interview.**	
6. Interviewee's Initials	|___|___|___| First letters of: first name, middle name and last name
7. Age	|___|___| Years
8. Occupation ?	□ Student □ Worker □ Unemployed □ Other ____________
9. Highest level of education completed (if studying?)	□ No education □ Primary □ Basic □ Middle □ Higher □ Basic Technical □ Middle Technical
10. Marital status?	□ Single □ Married □ Marital union □ Widow
11. Religion?	□ No Religion □ Christian - Catholic □ Christian - other □ Muslim □ Animist (traditional) □ Other Religion____________________________
12. Who do you live with?	□ Mother □ Father □ Father and Mother<br>□ Others _______________ ___________________________
13. Head of household	□ Mother □ Father □ Grandfather/grandmother<br>□ Others _____________________________
14. Reason/type of health problems that led you to come to the health facility today?	Describe<br>____
15. How did you come from home to the health facility?	□ Car □ Motorcycle taxi □ Bicycle taxi □ My bicycle □ My bicycle □ Hitchhiking □ On foot

16. How much money did you spend on transportation from home to the health facility (if applicable)?	Money in meticais ________<br>☐ Not applicable
17. How long did it take to walk from home to the health facility (if applicable)	Hours _______ and minutes _________
18. What time did you arrive here at the health facility?	Hours _______ and minutes _________
19. Set departure time (reminder)	Hours _______ and minutes _________

Now let's move on to questions about your knowledge and experience regarding the SRH and HIV services offered at this health facility.

Assessment of adolescent girls' knowledge and experience with the SRH and HIV services offered at the health facility

---

**1.**	In your opinion, what SRH and HIV services does the health facility offer to adolescent girls?<br>Note: Mark with X all applicable answers	
	a) Sexuality counseling	Yes ☐ No ☐
	b) Safe sex advice	Yes ☐ No ☐
	c) Pregnancy prevention advice	Yes ☐ No ☐
	d) Counseling on STI and HIV prevention	Yes ☐ No ☐
	e) Pregnancy test at SAAJ	Yes ☐ No ☐
	f) Test for STI/HIV diagnosis?	Yes ☐ No ☐
	g) VBG services?	Yes ☐ No ☐
	h) Prenatal consultation?	Yes ☐ No ☐
	i) Postpartum consultation?	Yes ☐ No ☐
	j) Abortion/post-abortion service?	Yes ☐ No ☐
		Yes ☐ No ☐
2.	What types of SRH and HIV services did you receive today at the health facility?	
	a) Sexuality counseling	Yes ☐ No ☐
	a) Safe sex advice	Yes ☐ No ☐
	b) Pregnancy prevention advice	Yes ☐ No ☐
	c) Counseling on STI and HIV prevention	Yes ☐ No ☐
	d) Pregnancy test at SAAJ	Yes ☐ No ☐
	e) Test for STI/HIV diagnosis?	Yes ☐ No ☐
	f) VBG services?	Yes ☐ No ☐
	g) Prenatal consultation	Yes ☐ No ☐
		Yes ☐ No ☐

	h) Postpartum consultation	Yes ☐ No ☐
	i) Post-abortion consultation?	Yes ☐ No ☐
	j) SRH and HIV pamphlet/leaflet	
3. What types of contraceptive methods did you receive during this visit to the health facility?		
	a) Male condom	Yes ☐ No ☐
	b) Female condom	Yes ☐ No ☐
	c) Pills	Yes ☐ No ☐
	d) IUD	Yes ☐ No ☐
	e) Implant	Yes ☐ No ☐
	f) Injectables	Yes ☐ No ☐
**4. How do you evaluate the environment of the health facility to serve adolescents?**		
	a) Is there a specific schedule for adolescents in this health facility?	Yes ☐ No ☐ I don't know ☐ Maybe ☐
	b) Does the health facility have a comfortable place for adolescents to sit?	Yes ☐ No ☐ I don't know ☐ Maybe ☐
	c) Does the health facility have a separate space to offer services to adolescents?	Yes ☐ No ☐ I don't know ☐ Maybe ☐
	d) Does the health facility have a separate waiting room for adolescents?	Yes ☐ No ☐ I don't know ☐ Maybe ☐
	e) Is there a counseling area for adolescents that provides privacy?	Yes ☐ No ☐ I don't know ☐ Maybe ☐
	f) Are adolescents greeted and served according to their needs or those of their partner(s)??	Yes ☐ No ☐ I don't know ☐ Maybe ☐
5. Level of satisfaction of adolescent girls with the SR&R and HIV services received		
	a) How satisfied were your needs today?<br>☐ Very satisfied<br>☐ Satisfied<br>☐ A little satisfied<br>☐ A little dissatisfied<br>☐ Unsatisfied<br>☐ Very dissatisfied<br>b) How do you evaluate the attitude of the provider who assisted you?<br>☐ Bad<br>☐ Acceptable<br>☐ Good<br>c) Overall, how do you evaluate the provision of SRH and HIV services in this health facility?<br>☐ Bad<br>☐ Acceptable<br>☐ Good<br>d) How satisfied were you that your needs were met today?<br>☐ Very satisfied	

	☐ Satisfied<br>☐ A little satisfied<br>☐ A little dissatisfied<br>☐ Unsatisfied<br>☐ Very dissatisfied

CONTACT INFORMATION: Would you mind if we contact you again if we have any follow-up questions? If you have any questions, our contact information is available on the participant information sheet/consent form we shared with you before we began this interview.

We have reached the end of the interview. Thank you for your attention, your time and your valuable contributions!
