# Supplementary material for: Knowledge and experiences of adolescent girls and young women in the use of sexual reproductive health and HIV services at health facilities in Maputo City, Mozambique
Source: Front Reprod Health. 2025 Nov 20;7:1667930. doi: 10.3389/frph.2025.1667930 (PMC12675394; doi:10.3389/frph.2025.1667930)
Supplement: Supplementary file 2 [file Table1.docx]

List of figure captions:

Fig 1. Knowledge of AGYW about SRH and HIV services offered

Fig 2. Types of SRH and HIV services received by the AGYW on the day of visit to the health facility
